# Systemic siRNA Nanoparticle-Based Drugs Combined with Radiofrequency Ablation for Cancer Therapy

**DOI:** 10.1371/journal.pone.0128910

**Published:** 2015-07-08

**Authors:** Muneeb Ahmed, Gaurav Kumar, Gemma Navarro, Yuanguo Wang, Svetlana Gourevitch, Marwan H. Moussa, Nir Rozenblum, Tatyana Levchenko, Eithan Galun, Vladimir P. Torchilin, S. Nahum Goldberg

**Affiliations:** 1 Laboratory for Minimally Invasive Tumor Therapies, Department of Radiology, Beth Israel Deaconess Medical Center/Harvard Medical School, 1 Deaconess Rd.–WCC-308B, Boston, Massachusetts, 02215, United States of America; 2 Department of Pharmaceutical Sciences and Center for Pharmaceutical Biotechnology and Nanomedicine, Northeastern University, 140 The Fenway, Boston, Massachusetts, 02115, United States of America; 3 The Goldyne Savad Institute of Gene Therapy, Hadassah Hebrew University Medical Center, Kiryat Hadassah POB 12000, Jerusalem, 91120, Israel; 4 Division of Image-guided Therapy and Interventional Oncology, Department of Radiology, Hadassah Hebrew University Medical Center, Kiryat Hadassah POB 12000, Jerusalem, 91120, Israel; National Yang-Ming University, TAIWAN

## Abstract

**Purpose:**

Radiofrequency thermal ablation (RFA) of hepatic and renal tumors can be accompanied by non-desired tumorigenesis in residual, untreated tumor. Here, we studied the use of micelle-encapsulated siRNA to suppress IL-6-mediated local and systemic secondary effects of RFA.

**Methods:**

We compared standardized hepatic or renal RFA (laparotomy, 1 cm active tip at 70±2°C for 5 min) and sham procedures without and with administration of 150nm micelle-like nanoparticle (MNP) anti-IL6 siRNA (DOPE-PEI conjugates, single IP dose 15 min post-RFA, C57Bl mouse:3.5 ug/100ml, Fisher 344 rat: 20ug/200ul), RFA/scrambled siRNA, and RFA/empty MNPs. Outcome measures included: local periablational cellular infiltration (α-SMA+ stellate cells), regional hepatocyte proliferation, serum/tissue IL-6 and VEGF levels at 6-72hr, and distant tumor growth, tumor proliferation (Ki-67) and microvascular density (MVD, CD34) in subcutaneous R3230 and MATBIII breast adenocarcinoma models at 7 days.

**Results:**

For liver RFA, adjuvant MNP anti-IL6 siRNA reduced RFA-induced increases in tissue IL-6 levels, α-SMA+ stellate cell infiltration, and regional hepatocyte proliferation to baseline (p<0.04, all comparisons). Moreover, adjuvant MNP anti-IL6- siRNA suppressed increased distant tumor growth and Ki-67 observed in R3230 and MATBIII tumors post hepatic RFA (p<0.01). Anti-IL6 siRNA also reduced RFA-induced elevation in VEGF and tumor MVD (p<0.01). Likewise, renal RFA-induced increases in serum IL-6 levels and distant R3230 tumor growth was suppressed with anti-IL6 siRNA (p<0.01).

**Conclusions:**

Adjuvant nanoparticle-encapsulated siRNA against IL-6 can be used to modulate local and regional effects of hepatic RFA to block potential unwanted pro-oncogenic effects of hepatic or renal RFA on distant tumor.

## Introduction

There has been much recent interest in using RNA interference (‘post-transcriptional gene silencing’) and particularly small interfering RNAs (siRNA) in cancer therapies. This has largely focused on either modulating cellular protein responses to improve efficacy of primary pharmacologic/oncologic therapies [1,2], augmenting anti-tumor immunity through selective knockdown of immune response suppressing proteins, or chemo-sensitization through silencing of growth factor receptor genes [3–6]. To overcome limitations in delivery with free siRNA in *in vivo* systems, siRNAs have been commonly administered using nanoparticle carriers (such as liposomes, micelles, or bound to particles) to maximize essentially *passive* tissue delivery (albeit in higher concentrations) to primary organs and target tumors [7–11]. Yet, even aided by nanocarrier delivery, challenges to intra-tumoral and targeted drug delivery persist [1,3]. Additionally, few studies have used adjuvant siRNA in conjunction with non-pharmacologic paradigms, such as modulating tissue or physiologic responses after surgical, interventional, or radiation treatments.

Image-guided thermal tissue ablation using radiofrequency, microwave, or laser energy to create local high temperature (60–100°C) heating around a percutaneously placed applicator is now in widespread clinical use to treat focal tumors of the liver, lung, kidney, and bone (>100,000 cases/year worldwide) [12]. Successful complete tumor ablation also requires the inclusion of a 5–10 mm rim of normal parenchyma as an ‘ablative margin’ to ensure complete eradication the tumor [13]. In an attempt to ensure completeness of treatment, we have shown that administration of even a single-dose of drug-loaded nanoparticle chemotherapy concurrently with RF ablation can lead to increases in local periablational drug delivery (up to 10-fold), with resultant increased tumor destruction, increased animal endpoint survival, and greater amounts of tumor destruction in patients with hepatic tumors [14–16]. Recently we have further successfully leveraged this improved focal delivery to target specific RFA-induced tissue responses (such as increased DNA intercalation or suppressing HSP production) [16,17].

A number of studies have demonstrated that sub-lethal lower tissue heating (40–47°C) around the ablation zone incites multiple secondary tissue reactions [18], including increased inflammatory cytokine (such as IL-6 and IL-10) [19] and growth factor (such as HGF, HIF-1α, and VEGF) [20–22] production; infiltration of scavenger and immunogenic cells into the periablational tissue [23]; and hyperthermia-induced increases in vascular permeability and endothelial leakiness [24]. In particular, recent studies have demonstrated that increased serum Interleukin-6 production after thermal ablation of normal liver (simulating the clinical endpoint of ablating the entire liver tumor and its ‘ablative margin’ of normal surrounding liver) is a key driver of periablational infiltration of inflammatory and immunogenic cells such as macrophages and α-smooth muscle actin (SMA) positive hepatic stellate cells [23,25]. These, in turn, can produce additional activation of downstream pro-growth pathways such as cytokines in the HGF/c-Met pathway, and increased hepatocyte proliferation in untreated liver—processes which are markedly reduced in IL-6 knockout mice [25]. Given that successful complete tumor ablation also requires the inclusion of a 5–10 mm rim of normal parenchyma as an ‘ablative margin’ to ensure complete eradication the tumor[13], studying these secondary systemic effects of RF ablation in normal organ tissue is imperative. The need for further study is supported by recent experimental studies demonstrating, ‘off-target’ stimulation of distant tumor growth after thermal ablation of liver and kidney tissue, and clinical studies that suggest a higher incidence of new HCC after hepatic RF ablation (approaching 80% at 5 years) compared to comparative un-treated cirrhotic patients (22–50%)[26–29]. Here, we build on our earlier experience using nanoparticle-mediated drug delivery preferentially targeted to the periablational rim to study whether polymeric micelle-like nanoparticles (MNPs) loaded with anti-IL6 siRNA can be used to suppress thermal ablation-induced IL-6 production, IL-6-mediated periablational cell infiltration, hepatocyte proliferation in untreated liver, and IL-6-mediated downstream RF ablation-induced stimulation of distant tumor growth.

## Materials and Methods

### Ethics statement

All animal studies were performed with the approval of the Beth Israel Deaconess Medical Center Institutional Animal Care and Use Committee and Hadassah Hebrew University Medical School Institutional Animal Care Ethics Committee.

### Animal models

For all experiments and procedures, anesthesia was induced with IP injection of a mixture of ketamine (50 mg/kg, Ketaject; Phoenix Pharmaceutical, St. Joseph, MO) and xylazine (5 mg/kg, Bayer, Shawnee Mission, KS). Animals were sacrificed with an overdose of carbon dioxide using SMARTBOX CO_2_ Chamber System (EZ systems, Palmer, PA) followed by cardiac puncture.

Experiments were performed in normal C57Bl mice (40±10g), or two breast adenocarcinoma cell lines (R3230 or MATBIII) implanted subcutaneously in female Fisher 344 rats (150±20g; 14–16 weeks old, Charles River, Wilmington, MA) [30]. The R3230 breast adenocarcinoma tumor line is a well-characterized line that we have been using for over 10 years [30]. The MATBIII tumor line was obtained from American Type Culture Collection (ATCC, Manassas, VA). Tumor implantation, evaluation, and preparation techniques were performed as previously described [30]. Briefly, one tumor was implanted into each animal by slowly injecting 0.3–0.4 mL of tumor suspension into the mammary fat pad of each animal via an 18-gauge needle. Tumors were measured every 1-2d until they reached 6–7 mm at which point they were included in studies.

### Application of RF ablation

Conventional monopolar RFA was applied by using a 500-kHz RFA generator (model 3E; Radionics, Burlington, MA), as has been previously described [30]. Briefly, the target organ was exposed via laparotomy using a subcostal (liver) or lateral (kidney) incision under sterile conditions while the animal was anesthetized. The 1-cm tip of a 21-gauge electrically insulated electrode (SMK electrode; Cosman Medical Inc, Burlington, MA) was placed in the liver or kidney. For animals treated with thermal ablation, RF energy was applied for 5 min with generator output titrated to maintain a designated tip temperature (70±2°C). This standardized method of RF application has been demonstrated previously to provide reproducible coagulation volumes with use of this conventional RFA system [30,31]. To complete the RF circuit, the animal was placed on a standardized metallic grounding pad (Radionics). For animals treated with either sham or an agent alone, the electrode was placed, but no energy was administered.

### Nanoparticle siRNA formulation

All materials were purchased from Sigma-Aldrich (St. Louis, MO) unless otherwise stated. Branched polyethylenimine (PEI) with a molecular weight of 1.8 kDa was purchased from Polysciences, Inc (Warrington, PA). 1,2-disrearoyl-sn-glycero-3-phosphoethanolamine-N-[methoxy (polyethylene glycol)-2000] (PEG-PE 2kDa) and 1,2-dioleoyl-sn-glycero-3-phosphoethanolamine-N-(glutaryl) (Glutaryl-PE) were purchased from Avanti Polar Lipids (Alabaster, AL). Nuclease-free water was purchased from Qiagen (Germantown, MD). All siRNA duplexes are from Dharmacon (Lafayette, CO). The synthesized sequences of siRNA targeting IL-6 were: 5’-AGUCGGAGGCUUAAUUACAdTdT-3’(sense), 5’-CAGGAAAUUUGCCUAUUGAdTdT-3’ (sense) and 5’-UAAGGACCAAGACCAUCCAdTdT-3’(sense)[32,33]. A scramble siRNA was used as negative control. The sequence of non-targeting control siRNA was 5’-AGUACUGCUUACGAUACGGdTdT-3’ (sense) [34].

Micelle-like nanoparticles were selected as the delivery vehicle based upon prior work demonstrating beneficial temporal delivery kinetics (6–12hr) and greater interstitial penetration in tissues surrounding the ablation zone [35]. We constructed micelle-like nanoparticles (MNPs) based on phospholipid modified-polyethylenimine conjugates (DOPE-PEI). The DOPE-PEI conjugate and the DOPE-PEI/siRNA complexes were prepared as previously described [34]. We have previously demonstrated that DOPE-PEI conjugates based on low molecular weight PEI self-assembled within micellar structures that condensed siRNA, showed high transfection efficiency and had a better toxicity profile than PEI 25 kDa and Lipofectamine (commonly used DNA/siRNA transfection reagents) [11]. We incorporated PEG-PE in the carriers to achieve an improved performance *in vivo*. The hydrophobic interactions between the lipid moieties in DOPE-PEI and those in PEG-PE resulted in their self-assembly into micelle-like particles with a mean size of 150 nm and provide some steric stabilization of complexes by forming a protective barrier and promoting increased stability *in vivo*. The DOPE-PEI/siRNA complexes were prepared by mixing equal volumes of DOPE-PEI with siRNA-IL-6 (equimolar pool of 3 sequences) or scramble siRNA to a final N/P ratio of 16. The siRNA solution was transferred to the polymer solution, mixed by vigorous pipetting and incubated for 15 min. The polymer/siRNA ratio was expressed as the nitrogen/phosphate (N/P) ratio, and calculated assuming that 43 g/mol corresponds to each repeating unit of PEI containing one amine and 316 g/mol corresponds to each repeating unit of siRNA containing one phosphate. Then, a previously freeze-dried lipid film of PEG-PE was hydrated with the complexes and allowed to stand at room temperature for 1h with intermittent shaking The PEG-PE2kDa:DOPE-PEI weight ratio was 2:1 [36]. Empty formulations were prepared by the hydration of the film with polymer solution only. Each mouse received a dose of 3.5 μg of siRNA in a final formulation volume of 100 μL. Each rat received a dose of 20 μg of siRNA in a final formulation volume of 200 μL. Each dose was administered by intraperitoneal injection (IP) at selected time points as specified above.

### Tumor harvesting

Animals were sacrificed at specified times outlined above using carbon dioxide euthanasia. The target tissue (primary site of ablation, untreated liver lobe, or distant tumor) was harvested, and sectioned perpendicularly to the direction of electrode insertion [17,30]. Distant tumors were also harvested and sectioned. All samples were fixed in 10% formalin overnight at 4°C, embedded in paraffin, and sectioned at a thickness of 5 μm. Tissues were stained with H&E for gross pathology.

### Quantification of IL-6 and VEGF levels

Serum and tissue levels of IL-6 (Mouse/M6000B and Rat /R6000B Quantikine kit, R&D Systems Inc., Minneapolis, MN) and VEGF (Rat/RRV00 Quantikine kit, R&D Systems) were determined using an enzyme-linked immunosorbent assay (ELISA) kit according to manufacturer’s instructions. Briefly, flash-frozen liver tissue was homogenized in a cold lysis buffer (Cell Signaling Technology Inc., Beverly, MA) consisting of a 0.1% proteinase inhibitor (Sigma-Aldrich). The homogenates were then centrifuged at 14,000 rpm for 20 min at 4°C, and the total protein concentration was determined using a bichinchoninic acid method (BCA) (Sigma-Aldrich). Undiluted serum was used. IL6 and VEGF values were then normalized to protein concentration. All samples and standards were measured in duplicate, and the average value was recorded as pg per mL [37,38].

### Immunohistochemical staining

Sections from ablated tissue and distant tumors were prepared and immunohistochemistry staining was performed for the following markers: α-SMA (hepatic stellate cells in the periablational rim), CDC-47 (hepatocyte proliferation, untreated liver) (has been previously described (Santa Cruz Biotechnology, Dallas, TX) [17,23]), Ki-67 (ab-16667 Abcam, Cambridge, MA) (tumor cell proliferation in distant tumor), and CD34 (microvascular density in distant tumor) (AF4117 R&D systems, Minneapolis, MN). Specimen slides were imaged and analyzed using a Micromaster I microscope (Fisher Scientific, Pittsburgh, PA) and Micron Imaging Software (Westover Scientific, Inc., Mill Greek, WA). Five random high power fields were analyzed for a minimum of 3 specimens for each parameter and scored in a blinded fashion to remove observer bias [16,17]. As an additional control to insure uniformity of staining, whenever direct comparisons were made, IHC was repeated with all relevant comparison slides stained at the same time.

### Statistical Analysis

The SPSS 13.0 software package (SPSS Inc., Chicago, IL) was used for statistical analysis. All data were provided as mean plus or minus SD. Selected (Day 0 and at the time of sacrifice) mean tumor sizes, and immunohistochemical quantification were compared using analysis of variance (ANOVA). Additional post-hoc analysis was performed with paired, two-tailed Student’s T-test, if and only if, the analysis of variance achieved statistical significance. A *P* value of less than 0.05 was considered significant. Tumor growth curves after treatment were analyzed using goodness-of-fit characterization. Mean post-treatment growth curve slopes plus or minus SD were also calculated and compared using ANOVA and paired, two-tailed T-tests.

## Results

### Nanoparticle anti-IL6 siRNA blocks local periablational and regional distant intrahepatic effects of thermal ablation

**Fig 1:**
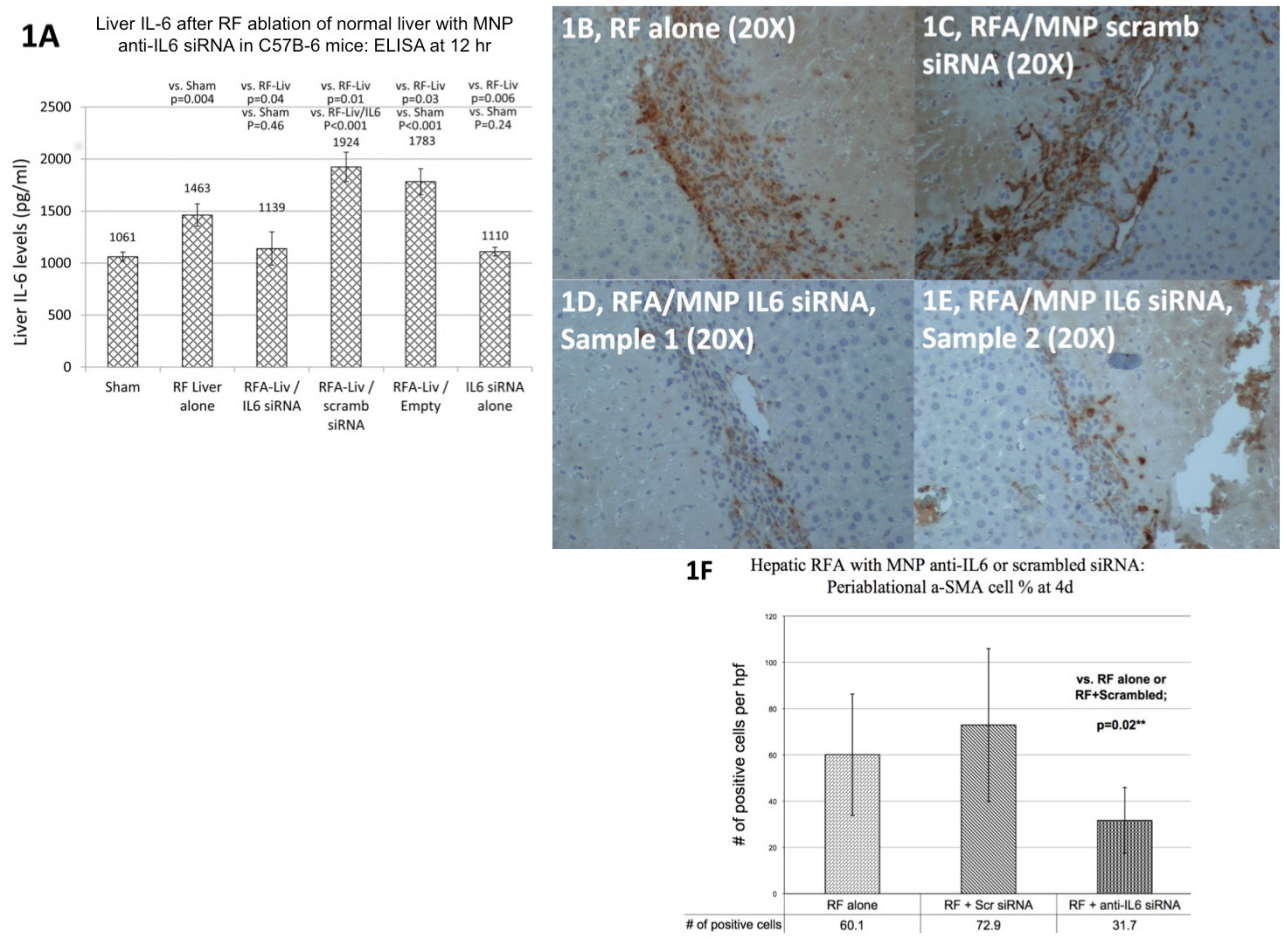
Hepatic thermal ablation increases liver IL-6 levels at 12hr post-treatment and periablational α-SMA+ activated myofibroblasts infiltration—which are suppressed with single dose adjuvant nanoparticle anti-IL6 siRNA. **(A)** Liver ELISA quantification of IL-6 levels 12hr after treatment (mean ± standard deviation). C57Bl mice were randomized to receive sham treatment, liver RF ablation, liver RFA / IP MNP anti-IL6 siRNA, MNP anti-IL6 siRNA alone, RFA / MNP scrambled siRNA, or RFA / empty carrier (n = 5–6 per group). Liver RFA increased local 12hr liver IL-6 levels (1,463±108 pg/ml) which was suppressed with adjuvant IP MNP anti-IL6 siRNA (1,139±159 pg/ml, p = 0.04). Additionally, adjuvant MNP anti-IL6 siRNA given 15 minutes after hepatic thermal ablation **(D, E)** in C57Bl mice suppressed periablational infiltration of α-SMA positive myofibroblasts compared to hepatic thermal ablation alone **(B)** or RFA combined with scrambled siRNA **(C) (F**, mean ± standard deviation, p<0.01).

Initial hepatic RF ablation studies were performed in normal C57Bl mice using standardized protocols to allow comparison to prior studies in normal and IL-6 knockout mice [25]. The following 6 treatment arms were compared (n = 5–6 animals/arm): hepatic RF thermal ablation alone (laparotomy and RF application for 5 min, tip temperature titrated to 70±2°C), sham procedure (laparotomy and needle placement without heating), RFA/MNP anti-IL6 siRNA (single dose of 3.5 μg siRNA given IP, 15 min post-ablation), RFA/MNP scrambled siRNA, MNP anti-IL6 siRNA alone, RFA/empty vehicle. Hepatic RF ablation increased local periablational tissue IL-6 levels compared to sham procedure at 12hr after treatment (1,463±108pg/ml vs. 1061±45pg/ml, p = 0.004, Fig 1A). Adjuvant MNP anti-IL6 siRNA given with hepatic RFA reduced local tissue IL-6 levels (1,139±159pg/ml; p = 0.04 vs. RFA alone, p = 0.46 vs. sham). Hepatic RFA/MNP scrambled siRNA increased local tissue IL-6 levels (1,924±141pg/ml; p<0.001 vs. sham, p = 0.01 vs. RFA alone, p = <0.001 vs. RFA/anti-IL6 siRNA). Tissue IL-6 levels after MNP anti-IL6 siRNA with sham treatment was 1,110±42pg/ml, and after RFA/empty carrier was 1783±125pg/ml.

As reported previously [23], standardized RF thermal ablation of normal liver resulted in infiltration of the periablational rim by α-SMA-positive activated myofibroblasts (also known as hepatic stellate cells, a surrogate marker for the complex periablational cellular infiltration that occurs after thermal ablation) at 4 days after treatment (60.1±26.3% positive cells/hpf within the rim, Fig 1B–1E). Adjuvant MNP anti-IL6 siRNA significantly reduced α-SMA positive periablational cellular infiltration compared to other treatment arms (31.7±14.3% positive cells/hpf vs. RF alone: 60.1±26.3%; RF/scrambled siRNA: 72.9±33.0%; p<0.001; Fig 1F). Both the sham procedure alone or combined with MNP anti-IL6 siRNA alone did not result in any increase in or other changes to periablational cellular infiltration.

**Fig 2:**
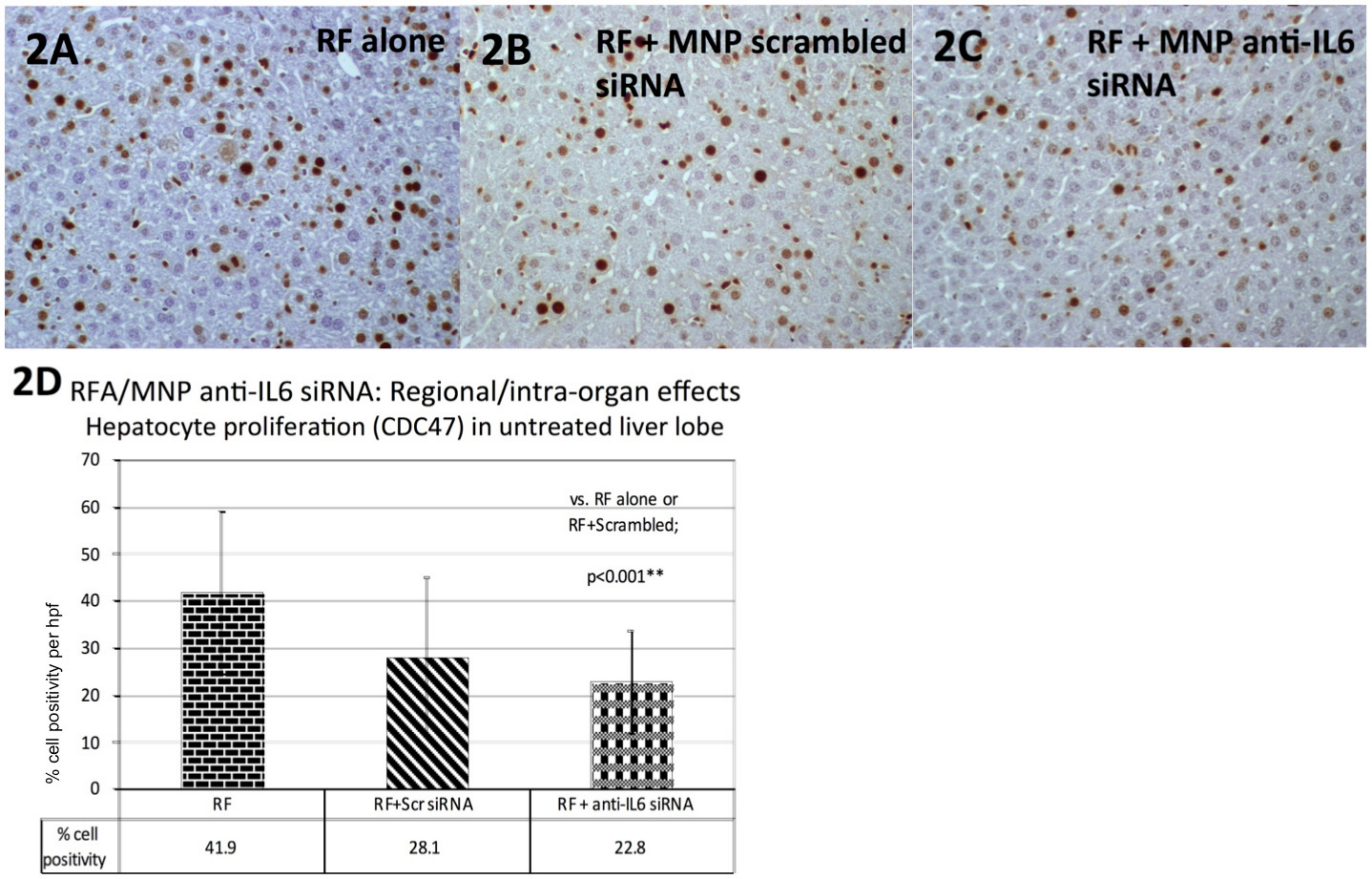
Adjuvant nanoparticle anti-IL6 siRNA suppresses thermal ablation-induced hepatocyte proliferation in the untreated, distant hepatic lobe. Adjuvant MNP anti-IL6 siRNA given 15 minutes after hepatic thermal ablation **(C)** in C57Bl mice (n = 5–6 animals/arm) suppressed hepatocyte proliferation in the distant, untreated liver lobe (with CDC47 staining, mean ± standard deviation) compared to hepatic thermal ablation alone **(A, D,** p<0.01). Hepatic thermal ablation combined with MNP scrambled siRNA was not significantly different from either thermal ablation alone or ablation combined with MNP anti-IL6 siRNA **(B, D)**.

Adjuvant MNP anti-IL6 siRNA also had organ-wide effects after hepatic thermal ablation. As shown by Rozenblum et al [25], thermal ablation of one lobe of the liver increased hepatocyte proliferation in the distant, untreated liver lobe, as measured by CDC47 staining (a marker of cells entering the G1 phase of cell replication) (42.016.2% positive cells/hpf, Fig 2). MNP anti-IL6 siRNA reduced the amount of hepatocyte proliferation compared to liver thermal ablation alone (23.0±10.7%/hpf vs. 42.016.2%/hpf, p<0.001). No difference was observed in hepatocyte proliferation in a distant, untreated liver lobe for RFA/MNP scrambled siRNA (28.1±16.7%/hpf) compared to RF ablation alone (p = 0.12) or RFA/MNP anti-IL6 siRNA (p = 0.15) [Fig 2A–2D].

### Nanoparticle anti-IL6 siRNA suppresses thermal ablation-induced stimulation of distant R3230 tumor growth and serum IL-6 levels after RF ablation of normal liver parenchyma

**Fig 3:**
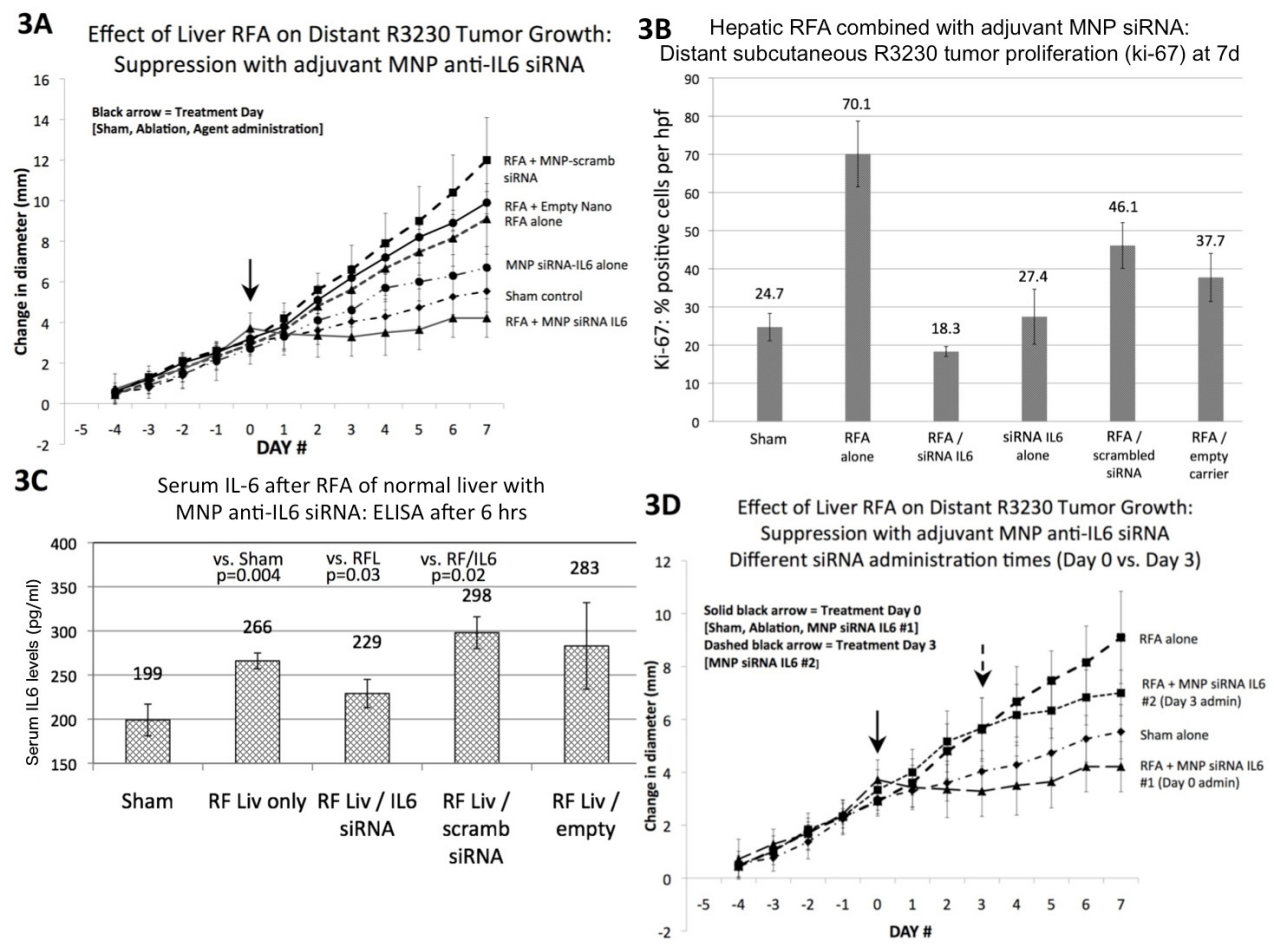
Hepatic thermal ablation-induced distant subcutaneous R3230 tumor growth is suppressed with adjuvant nanoparticle anti-IL6 siRNA. **(A)** Subcutaneous R3230 tumors implanted in Fisher 344 rats with similar growth rates were randomized at Day 0 to one of six different treatment arms (n = 6–7 animals/arm). Hepatic thermal ablation alone or combined with either empty carrier or MNP scrambled siRNA resulted in significantly greater tumor growth and change in diameter (5d before to 7d after treatment) compared to sham treatment (p<0.01 for all comparisons, mean ± standard deviation for all numbers presented). Adjuvant MNP anti-IL6 siRNA combined with thermal ablation resulted in distant tumor growth rate and end diameter that was the lowest of all treatment arms (p<0.01 for all comparisons). **(B)** Adjuvant MNP anti-IL6 siRNA combined with hepatic thermal ablation also reduced distant tumor proliferation (Ki-67) to sham levels compared to hepatic thermal ablation alone or combined with empty carrier or MNP scrambled siRNA (p<0.01 for relevant comparisons). **(C)** Hepatic thermal ablation alone or with MNP scrambled siRNA increased serum IL-6 levels at 6hr compared to the sham procedure (n = 3–4 animals/arm, p<0.02). This effect was suppressed with adjuvant MNP anti-IL6 siRNA (p = 0.03 vs. RF liver alone). **(D)** Comparison of the effect of siRNA administration timing showed that adjuvant MNP anti-IL6 siRNA administered at Day 0 resulted in the lowest post-treatment tumor growth rate and endpoint diameter (n = 3–4 animals/arm, p<0.05 for all comparisons). Adjuvant MNP anti-IL6 siRNA administered 3d post-ablation reduced the endpoint diameter compared to hepatic ablation alone, but was still significantly greater that either sham or combined-Day 0 treatment (p<0.05 for all comparisons).

To determine the impact on distant tumor growth to simulate the common condition of distant metastases, we used a subcutaneous breast tumor model (R3230) in which hepatic RF ablation has been associated with distant tumor growth [26]. Here, the following treatment arms were compared (n = 6–7 animals/arm): hepatic RF thermal ablation alone, sham procedure, RFA/MNP anti-IL6 siRNA (20 mcg of siRNA, IP delivery), RFA/MNP scrambled siRNA, MNP anti-IL6 siRNA alone, RFA/empty vehicle. The effect of these six treatment arms on distant subcutaneous tumor growth was assessed. All tumors grew at the same rate over 5d prior to randomization to various treatment arms. Thermal ablation of normal liver increased distant R3230 tumor growth for 7d after treatment compared to sham/control treatment (p<0.001; Fig 3A, Table 1). Adjuvant MNP anti-IL6 siRNA suppressed the thermal ablation-induced effects on distant R3230 tumor growth such that the mean tumor size at 7d with combination therapy was lower than either the sham (non-ablation) and hepatic RFA alone groups (p = 0.02 vs. sham, p<0.001 vs. hepatic RFA alone; Fig 3A, Table 1). Hepatic RF ablation combined with MNP scrambled siRNA resulted in the largest distant tumor diameter at 7d compared to all other treatment arms (19.3±1.7mm, p<0.03 for all comparisons).

**Table 1:** Evaluation of distant tumor after thermal ablation without and with adjuvant MNP siRNA.

Treatment arms	End tumor diameter (mm)	Change in diameter after treatment(mm)	Pre Growth Curve Slope	Post Growth Curve Slope	Tumor Ki-67 (% cell positivity/hpf)	MVD / CD34 (vessel # per hpf)
LIVER thermal ablation without and with anti-IL6 siRNA on distant R3230 tumor growth (7d)						
Sham treatment	13.8 ± 0.8	5.7 ± 1.1	0.64 ± 0.12	0.37 ± 0.16	24.7 ± 3.6	25.1 ± 8.1
RFA liver alone	17.0 ± 2.0	9.1 ± 1.7	0.62 ± 0.08	0.89 ± 0.24	70.1 ± 8.6	50.9 ± 15.9
RFA liver + anti-IL6 siRNA (given at Day 0)	12.6 ± 1.5	4.2 ± 1.0	0.75 ± 0.50	0.20 ± 0.01	18.3 ± 1.4	22.9 ± 3.2
anti-IL6 siRNA alone	14.7 ± 1.0	6.7 ± 1.0	0.59 ± 0.21	0.59 ± 0.18	27.4 ± 7.2	29.1 ± 1.0
RFA liver + scramb siRNA	19.3 ± 1.7	12.0 ± 2.1	0.65 ± 0.11	1.25 ± 0.30	37.8 ± 6.3	56.3 ± 3.1
RFA liver + empty carrier	17.1 ± 0.4	9.9 ± 0.5	0.65 ± 0.12	0.99 ± 0.09	46.1 ± 6.0	49.3 ± 7.8
RFA liver + anti-IL6 siRNA (given at Day 3)	15.5 ± 1.0	7.0 ± 0.9	0.85 ± 0.21	0.55 ± 0.49	28.2 ± 1.5	30.6 ± 2.8
KIDNEY thermal ablation without and with anti-IL6 siRNA on distant R3230 tumor growth (7d)						
Sham treatment	14.5 ± 1.2	6.4 ± 0.7	0.65 ± 0.11	0.45 ± 0.08	15.5 ± 5.6	16.9 ± 2.3
RFA kidney alone	19.6 ± 1.8	11.2 ± 1.5	0.61 ± 0.07	1.18 ± 0.26	45.1 ± 4.3	33.1 ± 4.4
RFA kidney + anti-IL6 siRNA	13.9 ± 1.2	5.7 ± 1.2	0.64 ± 0.22	0.35 ± 0.16	19.4 ± 2.0	19.6 ± 0.5
Anti-IL6 siRNA alone	15.7 ± 0.3	7.0 ± 0.9	0.75 ± 0.15	0.50 ± 0.16	28.4 ± 3.6	26.4 ± 0.9
LIVER thermal ablation without and with anti-IL6 siRNA in a SECOND TUMOR (MATBIII) (3.5d)						
Sham treatment	21.9 ± 1.2	11.4 ± 1.5	1.83 ± 0.21	0.39 ± 0.20	57.5 ± 7.7	32.8 ±7.9
RFA liver alone	24.4 ± 2.5	14.7 ± 2.0	1.86 ± 0.44	0.77 ± 0.20	75.8 ± 1.7	49.9 ± 7.3
RFA liver + anti-IL6 siRNA	20.6 ± 1.7	11.5 ± 1.0	1.80 ± 0.14	0.27 ± 0.22	44.8 ± 6.9	15.9 ± 0.9

All numbers provided as mean ± standard deviation.

Tumor proliferative index (Ki-67) in the distant R3230 subcutaneous tumor was measured for all treatment arms [Fig 3B, Table 1]. Hepatic thermal ablation increased distant tumor proliferation at 7d compared to the sham procedure (p = 0.001). Combination adjuvant MNP anti-IL6 siRNA and hepatic thermal ablation significantly reduced distant tumor proliferation (p = 0.045 vs. sham, p<0.001 vs. hepatic RFA alone). Thermal ablation combined with either empty carrier or MNP scrambled siRNA resulted in increased distant tumor prolilferation (p<0.04 compared to sham treatment). There was no difference between sham treatment and MNP anti-IL6 siRNA alone arms (p = 0.59).

Next, serum IL-6 levels were quantified by ELISA at 6hr after treatment for each of the six arms (n = 3–4 animals/arm) to determine the effect of adjuvant MNP anti-IL6 siRNA on ablation-induced systemic IL-6 levels. This time point was selected as serum IL-6 levels peaked at 6hr in this model post-hepatic RFA. Serum IL-6 levels were increased after thermal hepatic ablation (266±9pg/ml) compared to sham treatment (199±18pg/ml, p = 0.005) [Fig 3C]. The addition of MNP anti-IL6 siRNA reduced serum IL6 levels at 6hr (229±16pg/ml) compared to RFA alone (p = 0.03) or RFA/MNP scrambled siRNA (298±18pg/ml, p = 0.02). RFA/empty carrier also increased serum IL6 levels at 6hrs similar to RFA alone (298±18pg/ml, p = 0.57).

Finally, timing of siRNA administration was compared, and MNP anti-IL6 siRNA administered immediately after hepatic RFA (Day 0) resulted in complete suppression of RFA-induced tumor growth compared to administration at a later time point (3d) after hepatic RFA (Day 0 vs. Day 3, p<0.02) [Fig 3D, Table 1]. Similarly, for the hepatic RFA / nano anti-IL6 siRNA at Day 0, the tumor proliferative index at 7d was significantly lower compared to Day 3 administration (p = 0.001).

### Nanoparticle anti-IL6 siRNA suppresses hepatic ablation-induced distant tumor growth through reduction in VEGF-mediated tumor angiogenesis

**Fig 4:**
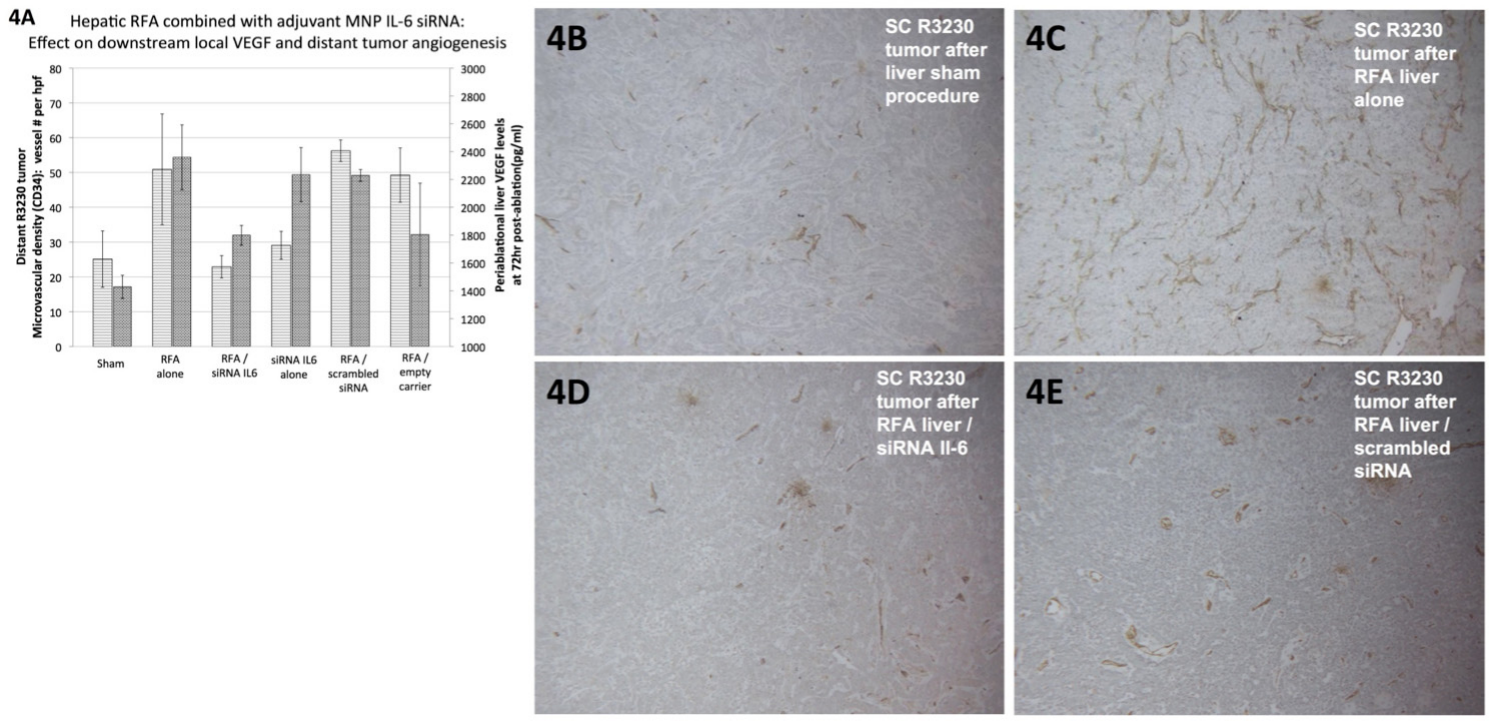
Adjuvant nanoparticle anti-IL6 siRNA suppresses hepatic ablation-induced distant subcutaneous R3230 tumor growth via reduction in VEGF-mediated angiogenesis. **(A)** Tissue levels of VEGF (Y-axis^2^, dark gray columns) in the periablational tissue surrounding the ablation zone were quantified at 72hr after different treatments (mean ± standard deviation, n = 3–4 animals/arm). Hepatic thermal ablation increased periablational VEGF levels, which were then suppressed with adjuvant MNP anti-IL6 siRNA (p<0.05 for all comparisons). **(B-E)** Similarly, hepatic thermal ablation led to increased microvascular density/angiogenesis (immunohistochemistry for CD34, A: Y-axis^1^, light gray columns) in distant subcutaneous R3230 tumor that was also suppressed with adjuvant anti-IL6 siRNA (n = 6–7 animals/arm).

Periablational liver tissue levels of VEGF were increased after thermal ablation, peaking at 72hr. Here, tissue VEGF levels in the periablational rim were compared using ELISA at 72hr after treatment for the following arms: hepatic RF thermal ablation alone, sham procedure, RFA/MNP anti-IL6 siRNA (20g of siRNA, IP delivery), RFA/MNP scrambled siRNA, MNP anti-IL6 siRNA alone, RFA/empty vehicle (n = 3–4 animals/arm). Hepatic ablation increased periablational tissue VEGF levels compared to sham treatment at 72hr (2359±233pg/ml vs. 1429±83pg/ml, p = 0.002) [Fig 4A]. Adjuvant MNP anti-IL6 siRNA reduced liver VEGF levels after thermal ablation (1799±71pg/ml, p = 0.02 vs. ablation). Hepatic ablation combined with adjuvant scrambled siRNA led to similar liver VEGF levels compared to RFA alone (2229±42pg/ml; vs. RFA alone: p = 0.51; vs. sham: p = 0.001). No difference was observed for thermal ablation combined with the empty carrier compared to other treatment arms (1804±370pg/ml; vs. RFA alone: p = 0.09; vs. sham: p = 0.16). MNP anti-IL6 siRNA alone had greater liver tissue VEGF levels compared to sham (2234±195pg/ml, p = 0.002).

Microvascular density (using CD34 staining) was then compared in distant subcutaneous tumors for each of the six treatment arms at 7d post-treatment [Fig 4B–4E, Table 1]. Hepatic thermal ablation increased distant tumor microvascular density at 7d compared to the sham procedure (p = 0.003). Combination adjuvant MNP anti-IL6 siRNA and hepatic thermal ablation significantly reduced distant tumor microvascular density (p = 0.01 vs. hepatic RFA alone). Thermal ablation combined with either empty carrier or scrambled siRNA resulted in increased distant tumor microvascular density (p<0.003 compared to sham treatment). There was no difference between sham treatment and MNP anti-IL6 siRNA alone arms (p = 0.46). These findings suggest that blocking of hepatic ablation-induced distant tumor growth by adjuvant anti-IL6 siRNA is mediated by suppression of downstream VEGF production and distant tumor angiogenesis.

### Nanoparticle anti-IL6 siRNA suppresses distant tumor growth after RF ablation in a second primary organ site (normal kidney)

**Fig 5:**
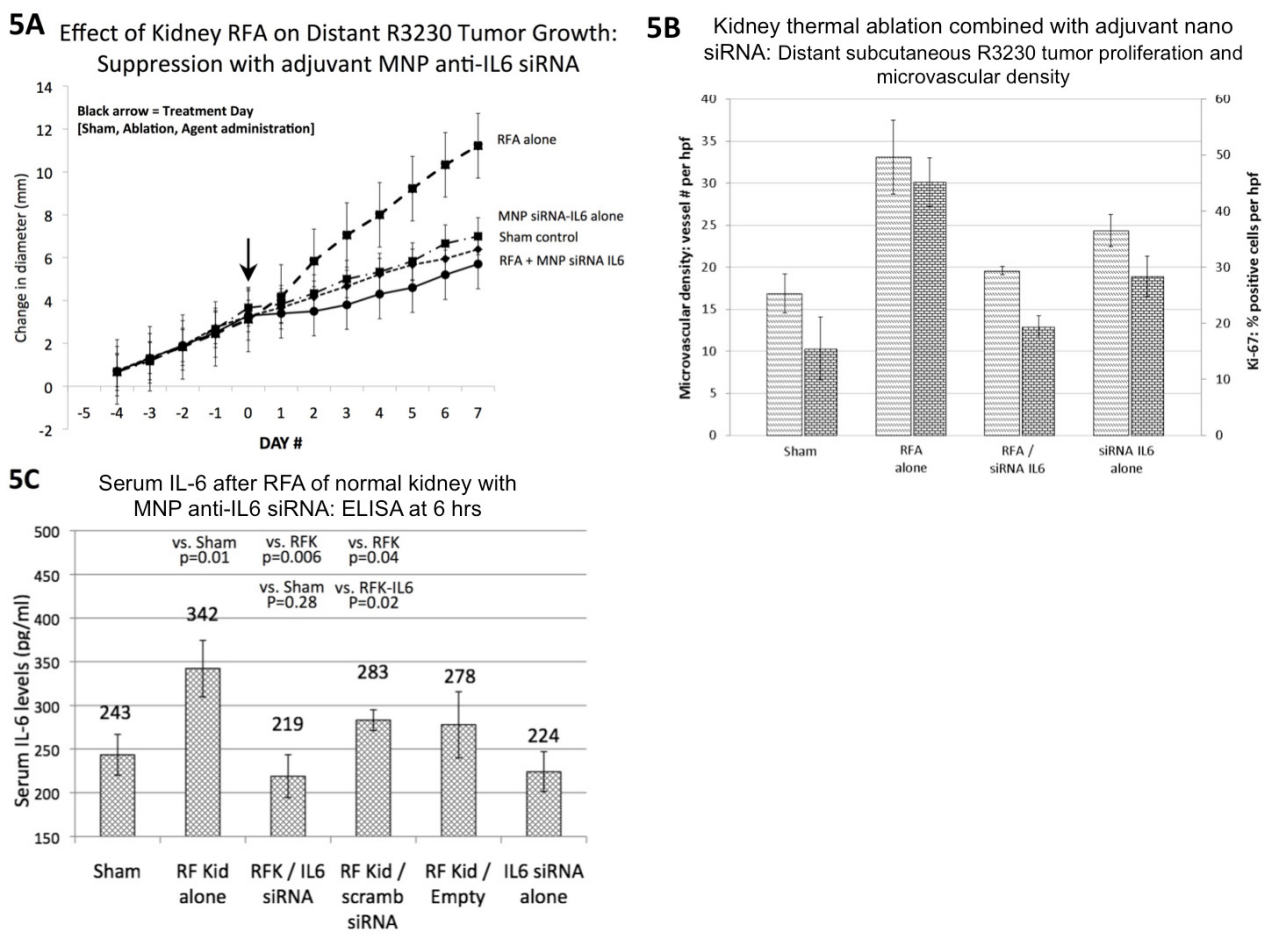
Thermal ablation of a second primary organ (kidney) leads to increased distant subcutaneous R3230 tumor growth that is suppressed with adjuvant nanoparticle anti-IL6 siRNA. **(A)** Subcutaneous R3230 tumors implanted in Fisher 344 rats with similar growth rates were randomized at Day 0 to one of four different treatment arms (n = 6–7 animals/arm). Thermal ablation of normal kidney alone resulted in significantly greater tumor growth and change in diameter (5d before to 7d after treatment) compared to sham treatment or MNP anti-IL6 siRNA alone (p<0.01 for all comparisons, mean ± standard deviation for all data). MNP anti-IL6 siRNA combined with thermal ablation reduced distant tumor growth rate and endpoint diameter to baseline sham levels. **(B)** Adjuvant MNP anti-IL6 siRNA combined with kidney thermal ablation also reduced distant tumor proliferation (Ki-67) and microvascular density (CD34) to baseline levels compared to kidney thermal ablation alone (p<0.01 for relevant comparisons). **(C)** Adjuvant MNP anti-IL6 siRNA suppressed kidney thermal ablation-induced elevations in serum IL-6 levels at 6hr post-treatment (n = 3–4 animals/arm, p = 0.006).

For tumor growth studies, kidney RF thermal ablation alone, sham procedure, RFA/MNP anti-IL6 siRNA (20g of siRNA, IP delivery), and MNP anti-IL6 siRNA alone were compared (n = 6–7 animals/arm). Similarly, RF ablation of normal kidney increased distant R3230 tumor growth compared to sham treatment that was also suppressed with single-dose adjuvant MNP anti-IL6 siRNA (given at Day 0) [Fig 5A, Table 1]. Tumor proliferative index and microvascular density for combination nanoparticle anti-IL6 and sham arms were equivalent to each other and lower compared to the group treated with RF ablation of normal kidney alone [Fig 5B, Table 1].

Next, serum IL6 levels were studied in the following treatment arms: kidney RF thermal ablation alone, sham procedure, RFA/MNP anti-IL6 siRNA (20g of siRNA, IP delivery), RFA/MNP scrambled siRNA, MNP anti-IL6 siRNA alone, RFA/empty vehicle (n = 3–4 animals/arm). Serum IL-6 levels (quantified by ELISA 6hr after thermal ablation) were increased after kidney thermal ablation (342±32pg/ml) compared to sham treatment (243±23pg/ml, p = 0.005) [Fig 5C]. The addition of MNP anti-IL6 siRNA reduced serum IL6 levels at 6hr (219±25pg/ml) compared to RFA alone (p = 0.03) or RFA/MNP scrambled siRNA (283±12pg/ml, p = 0.02). RFA/empty carrier also increased serum IL6 levels at 6hr similar to RFA alone (278±38pg/ml, p = 0.57).

### Nanoparticle anti-IL6 siRNA suppresses distant tumor growth in a second tumor model (MATBIII) after hepatic thermal ablation

**Fig 6:**
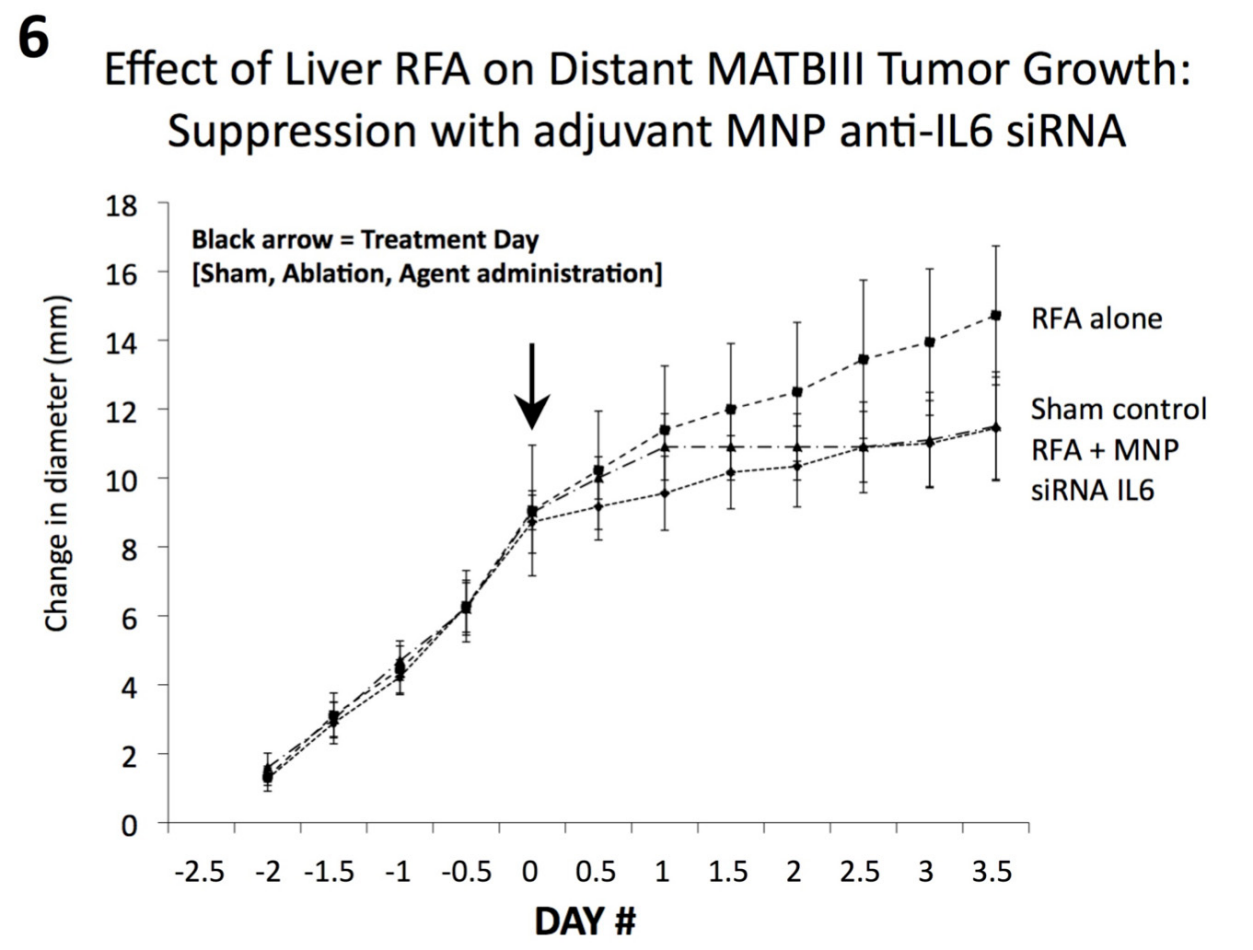
Hepatic thermal ablation-induced distant tumor growth is suppressed with adjuvant nanoparticle anti-IL6 siRNA in a second breast adenocarcinoma tumor model (MATBIII). Subcutaneous MATBIII tumors implanted in Fisher 344 rats with similar growth rates were randomized at Day 0 to one of three different treatment arms (sham treatment and hepatic RF ablation without and with adjuvant IP MNP anti-IL6 siRNA; n = 6–7 animals/arm). Given the rapid growth rate of this line, tumors were measured twice daily 2.5d pre- to 3.5d post-treatment (mean ± standard deviation). Hepatic thermal ablation resulted in a significantly larger endpoint tumor diameter compared to the sham group (p<0.05). Adjuvant MNP anti-IL6 siRNA suppressed the effects of hepatic thermal ablation, such that endpoint tumor diameter was equivalent to the sham group.

Next, the effect of MNP anti-IL6 siRNA on hepatic thermal ablation was assessed in a second subcutaneous tumor model (MATBIII rat breast adenocarcinoma line) at 3.5d post-treatment (due to the relatively faster innate tumor growth rate, all tumors reached the mandated size for sacrifice after 3.5d) [Table 1]. Hepatic thermal ablation without and with adjuvant IP MNP anti-IL6 siRNA was compared to sham treatment (n = 6–7 animals/arm). All tumors grew at the same rate over 2.5d prior to randomization to treatment. Hepatic thermal ablation again resulted in significantly larger distant subcutaneous MATBIII tumors at 3.5d compared to the sham procedure (p = 0.001, Fig 6). Adjuvant MNP anti-IL6 siRNA post-ablation reduced the tumor growth rate such that endpoint diameter was significantly smaller than hepatic ablation alone, and equal to the sham arm (vs. RFA alone p = 0.009, vs. sham p = 0.94, Table 1). RFA alone also resulted in significantly greater cellular proliferation and microvascular density in the distant MATBIII tumor compared to RFA with MNP anti-IL6 siRNA or sham treatment (p<0.05 for all comparisons, Table 1).

## Discussion

Several studies have reported that incomplete thermal tumor ablation of intrahepatic or renal tumors can have either stimulatory or suppressive effects on tumor cell growth in the partially injured residual cells present in periablational tissue or in intrahepatic/intra-organ tumor foci separate from the ablation site [21,22,39,40]. For example, Nijkamp et al. reported increases in periablative tumor outgrowth after incomplete ablation of murine intrahepatic colorectal metastases [21]. Regardless, potential RF ablation-induced stimulation of distant extrahepatic tumor growth remains poorly characterized. Here, we confirm recent reports that thermal ablation of normal organ parenchyma (such as liver or kidney) can stimulate distant tumor growth in at least two different tumor models [27], and further demonstrate that this in part due to ablation-induced increases in IL-6. Ultimately, this is potentially very clinically relevant, as the standard clinical endpoint in widespread practice is to ablate the entire tumor and up to a 5–10 mm circumferential margin of normal parenchymal tissue around the ablation zone [41]. Additionally, thermal ablation is increasingly applied as the primary treatment of oligometastatic disease, as part of multi-modality treatments where other existing tumors are being treated differently, or to target active metastatic foci in combination with systemic chemotherapy—all circumstances where small viable tumor cells are likely present separate from the ablation zone [42–44]. Therefore, for virtually all clinical cases of successful thermal tumor ablation, the potential for stimulation of tumor foci elsewhere in the body exists.

Our study further demonstrates that adjuvant nanoparticle siRNA can be successfully used to target IL-6 mediated locoregional and systemic effects of thermal ablation. Specifically, adjuvant siRNA can be used to suppress thermal ablation-induced increases in local and serum IL-6 cytokine levels, alter cellular infiltration patterns surrounding local thermal ablation, affect liver physiology/homeostasis by reducing ablation-induced hepatocyte proliferation in untreated liver, and achieve systemic suppression of un-intended ‘off-target’ distant pro-oncogenic effects of thermal ablation. Furthermore, we demonstrate that these RF-induced effects occur in various settings (here, in two tumor models, two organs, and two species), are IL6-mediated in these scenarios, and most importantly that they can be reproducibly modulated and attenuated with nano-delivered siRNA.

Thermal ablation incites several additional local tissue reactions, including increased oxidative and nitrative stress-mediated apoptosis and production of local tissue factors such as heat shock proteins, hepatocyte growth factor, and pro-angiogenic VEGF, HIF-1α, and PDGF [14,18,21,22]. Here, we also show that suppressing IL-6 with adjuvant siRNA blocks downstream production of VEGF and distant tumor angiogenesis, underscoring the potential utility of using adjuvant siRNA when the target is sufficiently upstream in the pathway. Additionally, while several of these pathways are linked to IL-6, those that are not could also potentially be targeted using other siRNAs to suppress production of alternative key proteins/mediators.

Our successful ‘proof-of-concept’ study demonstrates that nano-delivered siRNA can be used to suppress the secondary local and systemic ‘off-target’ effects (such as IL6-mediated inflammation) occurring after at least one clinically common procedure. Yet, there is also broader potential clinical applicability of this approach, as thermal ablative techniques have been incorporated into the treatment of a large number of diseases, including therapies such as pulmonary vein ablation for cardiac arrhythmias, esophageal ablation of Barrett’s esophagus, and in newer endovascular treatments for renovascular hypertension, mitral regurgitation, vascular stenosis, and venous reflux disease [45–48]. For several of these, secondary systemic inflammatory reactions (mediated by cytokines such as IL-6, C-reactive protein, and tumor necrosis factor) have been linked to treatment failure [49,50], and have been the basis for the incorporation of adjuvant peri-procedural anti-inflammatory drug therapies [51,52].

One of the main limitations of transitioning siRNA to therapeutic applications has been due to challenges in drug-delivery [53]. In particular, free siRNA has a very short blood circulating half-life (on the order of minutes to an hour), making target-site accumulation a critical barrier to siRNA efficacy [1,54]. Nanocarrier delivery has been one strategy studied to overcome obstacles of targeted delivery and extracellular siRNA breakdown by facilitating direct internalization through membrane fusion [55]. We have previously shown that nanocarriers preferentially accumulate around in the rim of tissue immediately surrounding the ablation zone due to heat-induced increases in vasodilation and endothelial leakiness [14,24,31]. Here, we show that thermal tissue ablation and nanocarrier anti-IL6 siRNA are particularly complementary as we take advantage of the fact that heating-induced vascular permeability occurs in the same geographic tissue region where infiltrating cells and increased IL-6 production is taking place, resulting in sufficiently high concentrations of anti-IL6 siRNA to render it effective in altering the cellular milieu and its composition. Given the predicTable and significant increases in nanoparticle delivery that can be achieved around tissue ablation or with targeted low-level hyperthermia, this combination strategy could potentially be used to facilitate siRNA delivery for other diseases that do not specifically require tissue heating but may benefit from higher levels of interstitial siRNA delivery. In addition to increased targeting specificity, additional advantages of this combination paradigm concentrating siRNA near cells that are the intended target (and conversely, minimizing exposure to non-target ‘normal’ cells), and increasing silencing potency by achieving higher local delivery.

We acknowledge several limitations in this study. Although we have focused on the pro-oncogenic effects of tumor ablation-induced inflammation, several studies have reported upon the potential for ablation-induced anti-tumor immunity (either alone or in combination with immunomodulation) with resultant tumor growth suppression [39,56–58]. While immunogenic effects of hepatic RF ablation are beyond the scope of this study, clearly additional research is required to understand the activating factors and balance between these two opposing effects. Thus, further translational study is envisioned defining the relevance of IL-6 and VEGF siRNA to other tumor types (including those with variable growth rates) and organs commonly ablated in clinical practice which may be more or less expressive of these cytokines. Indeed, given the host of reactions that have been identified in the periablational rim thus far, it is also likely that a number of other mediators are up-regulated (potentially downstream as part of the pathway studied here, or as a parallel pathway), such as other mediators of inflammation, the HGF/c-Met pathway, and downstream intracellular activators (such as PI3/Akt or STAT3 pathways). Several of these may also be potential therapeutic targets that merit further study. Along these lines, while we have studied radiofrequency-based thermal ablation (as this is the most commonly used modality in clinical practice), additional study will be required to determine the extent of applicability of our findings to other methods of ablation including, cryoablation, microwave thermal ablation, and irreversible electroporation[12]. Indeed, even higher post-treatment IL-6 levels have been reported for some therapies, such as cryoablation, compared to RF ablation [19]. Finally, it is possible that administration of the adjuvant drug, either through siRNA alone, interaction of lipid carrier components in the periablational rim, or the act of intraperitoneal administration also contributes to the inflammatory response. Indeed, we have previously shown that lipid components of the nanoparticle can be active independent from the drug payload [59]. Similarly, siRNA can induce a reactive inflammatory response [60], which likely explains the marginally higher distant tumor growth rate in the scrambled siRNA treatment arm (where there are no suppressive effects of the anti-IL6 siRNA) compared to even RF ablation alone. However, ultimately, the use of adjuvant anti-IL6 siRNA with RF ablation effectively suppresses any additional increases in inflammation associated with its administration.

In conclusion, we demonstrate that thermal ablation of normal organ parenchyma (such as is required in clinical practice) can result in Interleukin-6-mediated locoregional and systemic effects (including distant tumor growth). We further show that the novel paradigm of combining adjuvant nanoparticle anti-IL6 siRNA can be successfully used to suppress potentially pro-oncogenic effects of thermal ablation.
